# Supplementary material for: Precision screening identifies mitoxantrone as a multitarget inhibitor in ageing-associated cancers with extensive computational validation and doxorubicin comparison
Source: PLoS One. 2026 Feb 13;21(2):e0341446. doi: 10.1371/journal.pone.0341446 (PMC12904431; doi:10.1371/journal.pone.0341446)
Supplement: Attachment — Submitted filename: Rebuttal_letter_PlosOne.pdf [file pone.0341446.s003.pdf]

Additional Editor's Comments

One of the reviewers has suggested citations that may not be directly relevant to the manuscript. The authors are encouraged to decline such recommendations, ensuring that all referenced works remain closely aligned with your research contributions. Declining these suggested citations will not affect the editorial decision on the manuscript.

Dear Editor, many thanks for your comment. We have only included the relevant references.

Comments to the Author

1. Is the manuscript technically sound, and do the data support the conclusions? The manuscript must describe a technically sound piece of scientific research with data that supports the conclusions. Experiments must have been conducted rigorously, with appropriate controls, replication, and sample sizes. The conclusions must be drawn appropriately based on the data presented.

Reviewer #1: Yes

Reviewer #2: Yes

Dear reviewer, many thanks for appreciating our work.

---

2. Has the statistical analysis been performed appropriately and rigorously?

Reviewer #1: N/A

Reviewer #2: Yes

Dear reviewer, many thanks for appreciating our work.

---

3. Have the authors made all data underlying the findings in their manuscript fully available? The PLOS Data policy requires authors to make all data underlying the findings described in their manuscript fully available without restriction, with rare exception (please refer to the Data Availability Statement in the manuscript PDF file). The data should be provided as part of the manuscript or its

supporting information, or deposited to a public repository. For example, in addition to summary statistics, the data points behind means, medians and variance measures should be available. If there are restrictions on publicly sharing data—e.g. participant privacy or use of data from a third party—those must be specified.

Reviewer #1: Yes

Reviewer #2: Yes

Dear reviewer, many thanks for appreciating our work, and we have now also cited the supplementary sheets to make it better.

---

4. Is the manuscript presented in an intelligible fashion and written in standard English? PLOS ONE does not copyedit accepted manuscripts, so the language in submitted articles must be clear, correct, and unambiguous. Any typographical or grammatical errors should be corrected at revision, so please note any specific errors here.

Reviewer #1: Yes

Reviewer #2: Yes

Dear reviewer, many thanks for appreciating the English language of the work.

---

5. Review Comments to the Author

Please use the space provided to explain your answers to the questions above. You may also include additional comments for the author, including concerns about dual publication, research ethics, or publication ethics. (Please upload your review as an attachment if it exceeds 20,000 characters)

Reviewer #1:

Dear Authors, I read your manuscript, and it is wonderful. The work is very interesting, and at the same time, the way you presented figures, tables, and formatted the manuscript made it very easy to read and follow. However, there are still some changes or improvements that you need to make before it is accepted.

Dear reviewer, many thanks for appreciating our teamwork. We have now tried to make our manuscript better and addressed each of your comments.

1. Mention the number of compounds taken for the docking in the Abstract, and do not make keywords go in italics.

   Dear reviewer, we have now added the text in the abstract to make it clearer and updated the keywords.

2. Provide the list of abbreviations at the end of the manuscript in a single subheading.

   Dear reviewer, we have now provided the list of abbreviations at the end of the manuscript.

3. Add a dedicated heading for the limitations and future directions of the work.

   Dear reviewer, we have now added a dedicated heading for the limitations and future direction of the work.

4. The conclusion has too much data to understand. Can you make the line 1096-1100 a bit smaller and clearer?

   Dear reviewer, we have now made it less dense.

5. Funding and Acknowledgements should be in different points.

   Dear reviewer, we have now made the funding and acknowledgement in separate headings.

6. The supplementary file has too much data to report, but it is not cited in the manuscript. Authors are also recommended to cite the sheets.

   Dear reviewer, we have now cited the supplementary sheets in the manuscript.

7. RMSD, RMSF, and other abbreviations are explained multiple times. Keep the full form only at the first occurrence, then use the shorter form thereafter.

   Dear reviewer, we have now updated it throughout the manuscript.

   In my opinion, after addressing the above points, the manuscript is ready for acceptance.

   Dear reviewer, many thanks for appreciating our work and marking it for acceptance.

Reviewer #2:

1. Compare the HOMO-LUMO gap energy with biologically active molecules in the literature (10.1134/S0036024425701249, 10.1080/00387010.2024.2432331, 10.55730/1300-0527.3702).

Dear reviewer, we have now compared the energy for HOMO-LUMO and added relevant references to support the context.

2. Support the active site found by molecular docking and molecular dynamics calculations with experiments or literature.

   Dear reviewer, we used the native co-crystallised ligand binding sites of each protein to generate the docking grids, which represent experimentally validated active sites. The key residues identified in our docking and MD analyses are also consistent with previously reported binding-site residues in the literature.

---

6. PLOS authors have the option to publish the peer review history of their article (what does this mean?). If published, this will include your full peer review and any attached files. If you choose "no", your identity will remain anonymous but your review may still be made public. **Do you want your identity to be public for this peer review?** For information about this choice, including consent withdrawal, please see our Privacy Policy.

Reviewer #1: No

Reviewer #2: **Yes:** Sefa Celik

Dear anonymous reviewer and Dr Celik, many thanks for carefully reviewing our work and appreciating it. We look forward to getting your feedback for acceptance or revision if there are still some points that need to be addressed.

Dear Editor and Reviewers, we would like to thank you for providing us with suggestions and the opportunity to revise the manuscript. We have addressed all the comments and responded above to each of your comments accordingly.
